# nTMS in spinal cord injury: Current evidence, challenges and a future direction

**DOI:** 10.1016/j.bas.2025.104234

**Published:** 2025-03-14

**Authors:** Josephine Jung, Sabina Patel, Azharul Khan, Alba Diaz Baamonde, Ana Mirallave-Pescador, Yasir A. Chowdhury, David Bell, Irfan Malik, Nick Thomas, Gordan Grahovac, Francesco Vergani, Aminul I. Ahmed, José Pedro Lavrador

**Affiliations:** a Institute of Psychiatry, Psychology & Neuroscience, King’s College London, UK; b Neurosciences Clinical Trials Unit, King’s College Hospital NHS Foundation Trust, London, UK; c Department of Neurosurgery, King’s College Hospital NHS Foundation Trust, London, UK; d Department of Neurophysiology, King’s College Hospital NHS Foundation Trust, London, UK

**Keywords:** Spinal cord injury, Transcranial magnetic stimulation, Rehabilitation

## Abstract

Spinal Cord Injury (SCI) has devastating consequences for patients and their families. Over the last few decades, a renewed interest in the utilization of non-invasive and cost-effective therapeutic technologies in the management of patients with SCI has emerged. This includes stimulation with navigated transcranial magnetic stimulation (nTMS) in order to improve the outcome for these patients alongside with existing clinical tools. nTMS has shown encouraging preliminary results in both clinical assessment and rehabilitation (motor and pain) of patients with SCI. However, different protocols – stimulation parameters, length of treatment and combination with other modalities – and patient selection criteria hampered definitive conclusions. So far, none of these have been adapted in regular clinical practice. In this article, we provide an overview on different assessment and therapeutic strategies using nTMS and review their effectiveness.

## Highlights

• Spinal Cord Injury (SCI) has devastating consequences for patients and their families.
• nTMS has shown encouraging results in clinical assessment and rehabilitation of SCI.
• We provide an overview on assessment and therapeutic strategies using nTMS in SCI.

## 1 Introduction

Acute spinal cord injury (SCI) is a devastating condition that has an incidence between 2500 and 4400 new cases per year in the UK with an estimated 50,000 to 105,000 people currently living with SCI in the UK (Patek and Stewart, 2023; https://www.spinal.co.uk/news/new-spinal-cord-injury-data-revealed/.SpinalInjuriesAssociation-SIAHouse,2TruemanPlace, Oldbrook, 2024). Lifetime costs for these cases has been conservatively estimated as £1.43 billion with a mean of £1.12 million per SCI case (McDaid et al., 2019).

SCI can be categorised into traumatic – the most common, and non-traumatic SCI. The most common causes for traumatic SCI are high-speed road traffic accidents, falls which can be relatively low-impact in the elderly population, and sports injuries. Common causes for non-traumatic SCI include metastatic spinal cord compression, cord infarction, or compression by a mass lesion such as epidural haematoma, abscess or tumour (https://www.spinal.co.uk/news/new-spinal-cord-injury-data-revealed/.SpinalInjuriesAssociation-SIAHouse,2TruemanPlace, Oldbrook, 2024).

Similarly to traumatic brain injuries, the goal in management of the acute trauma to the spine is to prevent secondary injury (Patek and Stewart, 2023). The primary injury to the cord, either by transient or persistent cord compression or distraction/transection, will have taken place by the time the patient arrives to the acute trauma centre.

The pathophysiology of SCI can be divided into complete and incomplete injury according to the American Spinal Injury Association (ASIA) Impairment Scale (Injury, 1982). Complete injury (ASIA A) means there is no motor or sensory function below the level of injury and is associated with the poorest outcome. Most SCIs however are incomplete (ASIA B-D) (Shulga et al., 2021a; Ackery et al., 2004; Chen and DeVivo, 2019; Fawcett et al., 2007) and as such there remains a degree of function below the level of injury which can be either motor and/or sensory and incorporates the sacral elements. This is associated with a better recovery outcome and lower mortality than complete injury. There have been studies trying to quantify the degree of neurological recovery in ASIA A injury which is around 5 % (half of which will recover to ASIA C and the other half to ASIA D). On the contrary, 50 % of patients with incomplete injury will recover one or more ASIA levels (Scivoletto et al., 2014).

The treatment focus in SCI has been put on the acute management including neuroprotection to prevent secondary injury, as well as long term management including repair and regeneration, cell-based therapies and neuroplasticity (Fischer et al., 2024; Punjani et al., 2023). One of these novel non-invasive technologies is navigated transcranial stimulation (nTMS) which has been used in rehabilitation to induce neuroplasticity. nTMS uses a figure-of-eight coil combined with E-field navigation to deliver a magnetic pulse to the motor cortex and elicit a motor evoked potential (MEP) in the hand or lower limb on the contralateral side (Jung et al., 2019). It calculates the strength, location, and direction of the stimulating electric field into the cortical tissue (J and R, 2002). Estimates of the induced electric field are based on a dynamic spherical model adjusted in real time and on physical stimulation parameters (Sarvas, 1987; Tarkiainen et al., 2003).

While other more invasive strategies such as gene therapy or mesenchymal stem cell transplants have had promising results with some showing an improvement in ASIA score of up to 75 %, most of these studies are Phase 1 or 2 trials, and focus on ASIA A patients only which does not represent the majority of SCI (Yamazaki et al., 2020). Other more invasive strategies include neurostimulation via deep brain stimulation or spinal cord stimulation have failed to give meaningful functional results with regards to motor movement and sensation, however they have found application for the management of neuropathic pain post SCI and the treatment of the neuropathic bladder (Chari et al., 2017). None of these have translated so far into clinical practice, albeit that ARC^EX^ therapy, a non-invasive spinal cord electrical stimulation tool, has received FDA approval for roll-out in the US. (Moritz et al., 2024) In this review we aim to explore the current state of play of nTMS in rehabilitation for SCI and current evidence from clinical trials. Furthermore, we would like to explore its role in diagnosis and prognostication of traumatic and non-traumatic SCI to identify intact anatomy and provide an outlook into potential future applications.

## 2 Outcome measurements

**Fig. 1:**
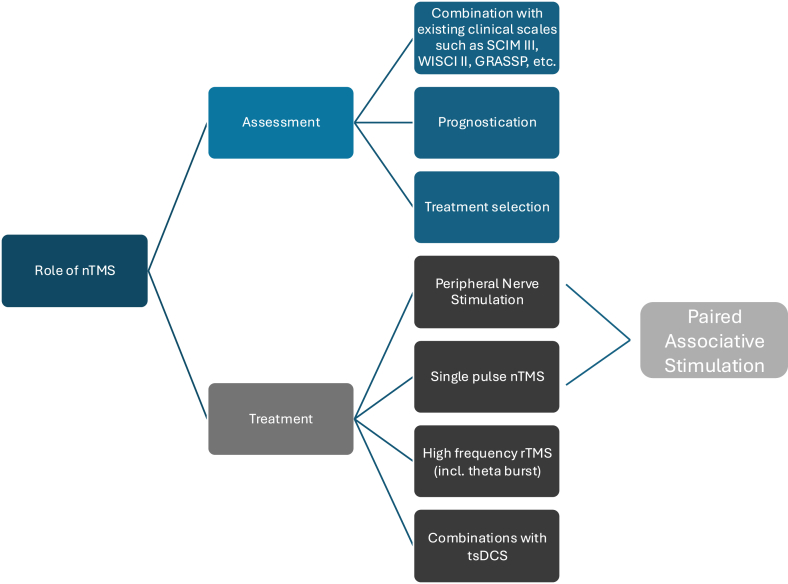
Overview on treatment of spinal cord injury.

nTMS can be used in assessment and treatment of patients with both traumatic and non-traumatic SCI (Fig. 1). When it is being used in the assessment of SCI it can provide objective outcome measures that can add to the existing scales that are currently being used. While the ASIA score can be a useful tool in assessing the injury itself and be used for prognostication, outcome measures must be practical, reproducible and granular. The most used assessments are the A) Spinal Cord Independence Measure (SCIM) III score – a self-assessment multiple choice questionnaire (scored out of a 100) validated for spinal cord injury patients, which includes questions evaluating self-care, breathing, sphincter dysfunction and mobility; B) Walking Index for Spinal Cord Injury (WISCI) II – a validated scale that measures type and amount of assistance required for spinal cord injury patients to mobilise and is often performed alongside assessments such as the 10 m walking test and timed-up-and-go test; C) Graded Redefined Assessment of Strength Sensibility and Prehension (GRASSP) – a clinical impairment measure assessing upper limb function in tetraplegic patients. Its domains of physical assessment include motor function, sensation and prehension (cervical injury only) (Itzkovich et al., 2018; Ditunno et al., 2013; Kalsi-Ryan et al., 2012). There is an argument to be made to overhaul these assessments into ones that are more meaningful for patients without losing their validity. One may speculate that the correlation of the above currently available scales may provide insight about the potential for nTMS in prognostication for recovery and patient selection for appropriate novel treatments.

### 2.1 TMS modalities and strategies

**Fig. 2:**
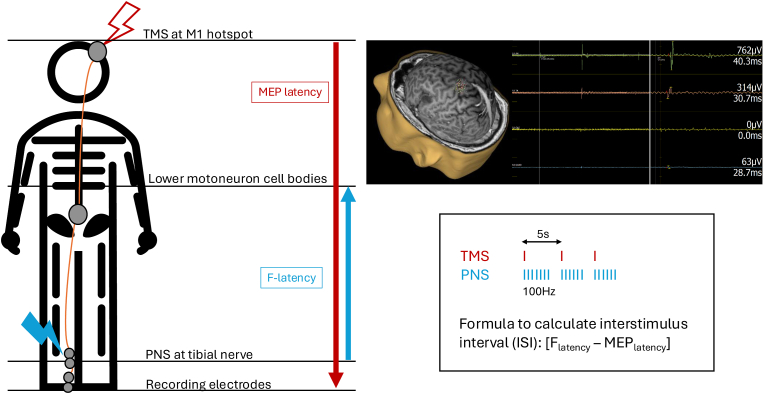
Overview on Paired Associative Stimulation (PAS). Stimulation of the tibial nerve (blue lightning) and M1 hotspot over the motor cortex (red lightning). The interstimulus interval between TMS and peripheral nerve stimulation (PNS) is calculated prior to stimulation using the F-latency and MEP latency.

While high-frequency repetitive TMS (which consists usually of a long train of pulses at 100 % resting motor threshold (RMT) with a pause between trains to prevent overheating and seizure induction), has been used in the treatment of depression ((Q and uality), 2021; Janicak and Dokucu, 2015; Morriss et al., 2024; Berlim et al., 2014; Goodman et al., 2024) and neuropathic pain (including SCI-associated neuropathic pain) with reasonable success (Gao et al., 2017), there has been limited evidence on the use of rTMS for SCI. In fact, Krogh et al. (2022) reported that the use of rTMS has no short-term recovery effect and any potential effect on long-term recovery is likely to be related to a task-specific training effect. In their randomised controlled trial there was no impact of rTMS on regaining gait function. Newer studies use a combination of nTMS (single pulse) with peripheral nerve stimulation (PNS), so called high-frequency paired associative stimulation (PAS), in order to potentiate the MEP and promote neuroplasticity (Shulga et al., 2016a, 2016b, 2021a; Tolmacheva et al., 2017, 2019). The interstimulus interval (ISI) is calculated using the formula: [F_latency_ -MEP_latency_], which are both measured prior to the PAS session (Fig. 2). In spinal PAS, as opposed to cortical stimulation, the orthodromic and antidromic volleys elicited by nTMS and PNS coincide at corticomotoneuronal synapses at the spinal cord leading to a long-term potentiation-like effect (Shulga et al., 2016b). This has been shown to lead to significant increase in independence (Rodionov et al., 2019).

In addition to different treatment modalities, different stimulation coils can be used which vary in their penetration depths and stimulation ranges. There are circular coils with zero intensity in the centre and figure-of-eight coils which have their highest intensity in the centre. As opposed to circular coils which have a wide stimulation range, figure-of-eight coils provide a more focussed stimulation (Wang et al., 2023).

In summary, there are a plethora of treatment coils, stimulation protocols and outcome measures in use which can be utilised for the application of TMS in rehabilitation of SCI injuries.

### 2.2 Current evidence for the use of TMS in SCI for rehabilitation

Level 1 evidence for the use of TMS in SCI rehabilitation is scarce. An overview on randomised controlled trials can be found in Table 1 (Krogh et al., 2022; Kuppuswamy et al., 2011; Benito et al., 2012; Gharooni et al., 2018; Jo and Perez, 2020; Adeel et al., 2022; Lin et al., 2023; Nogueira et al., 2024; Belci et al., 2004), two study protocols only have been listed in Table 2 (de Araújo et al., 2017; Sun et al., 2022). The number of patients included in each study varies between 11 and 115, indicating that some of these are lacking in power for statistical analysis. Most of the studies focussed on incomplete and traumatic SCIs. There is great variability in the TMS protocol used (stimulation varies between 4 weeks and 8 weeks and type of stimulation applied is vastly different (paired associative stimulation (PAS), vs intermittent theta-burst (iTBS) vs combinations with trans-spinal direct current stimulation (tsDCS)). Some of these studies show promising results particularly with regards to improvements in lower extremity motor score (LEMS) such as Benito et al. (2012) and Krogh et al. (2022), albeit not significant in the latter study. Benito et al. (2012) analysed data of 17 patients in a randomised double-blinded RCT who underwent either rTMS or SHAM stimulation. They were able to show a significant improvement in LEMS, Modified Ashworth Score (MAS), 10 m walking test, cadence, step length, and Timed Up and Go test in the stimulation group compared to SHAM group. However, the majority of their patients had thoracic injuries and all of them were ASIA D inherently carrying a better prognosis than ASIA A-C. Others focussed on spasticity like Gharooni et al. (2018) who were able to demonstrate that iTBS was able to significantly reduce spasticity on the MAS and the Visual Analogue Scale for spasticity, however the authors acknowledged the lack of neurophysiological assessments, a small sample size (8 complete data sets) and low test-retest reliability.

**Table 1:** List of randomised controlled trials for the use of rehabilitative TMS in spinal cord injury.

Author(s), Journal and Year	Title	Patients (N)	SCI grade	Type of SCI	Stimulation type	Functional outcomes
Kuppuswamy et al., Clinical Neurophysiology 2011 (Kuppuswamy et al., 2011)	Action of 5 Hz repetitive transcranial magnetic stimulation on sensory, motor and autonomic function in human spinal cord injury	N = 15	ASIA A-D	Mostly traumatic, one patient non-traumatic	rTMS + SHAM	Single-blinded study. rTMS treatment only lasted 5 days. No difference in Action Research Arm Test (ARAT), Peg-board test, ASIA scores, upper limb motor scores. Follow up completed at 12 days.
Benito et al., Top Spinal Cord Inj Rehabil 2012 (Benito et al., 2012)	Motor and Gait Improvement in Patients With Incomplete Spinal Cord Injury Induced by High-Frequency Repetitive Transcranial Magnetic Stimulation	N = 17	ASIA D only	Traumatic and non-traumatic SCI	rTMS vs SHAM	Improvement in lower extremity motor score (LEMS), spasticity, and gait following 15 daily sessions of real rTMS in patients with incomplete SCI. Maintained functional gains for a minimum of 2 weeks.
Gharooni et al., Spinal Cord 2018 (Gharooni et al., 2018)	Intermittent theta-burst stimulation for upper-limb dysfunction and spasticity in spinal cord injury: a single-blind randomized feasibility study	N = 12	ASIA B-D	Mostly traumatic, two patients with non-traumatic	iTBS vs SHAM	Mostly looked at effects on spasticity and not functional outcomes, SHAM control was easily identified, no double blinding.
Jin Jo H and Perez MA, Brain 2020 (Jo and Perez, 2020)	Corticospinal-motor neuronal plasticity promotes exercise-mediated recovery in humans with spinal cord injury	N = 38	ASIA A-D	Mostly traumatic, one patient with non-traumatic SCI	PCMS vs SHAM	Decreased time-to-perform tasks in paired- corticospinal-motor neuronal stimulation (PCMS) group, which remained sustained after 6 months.
Adeel et al., Journal of the Formosan Medical Association2022 (Adeel et al., 2022)	Effects of paired stimulation with specific waveforms on cortical and spinal plasticity in subjects with a chronic spinal cord injury	N = 10	ASIA B-D	Traumatic SCI, two patients with postsurgical SCI	rTMS + tsDCS vs iTBS + tsDCS vs SHAM + SHAM	Single-blinded study. Significant improvement in LEMS with both stimulation groups after 5 days of treatment. No follow-up.
Krogh et al., Spinal Cord 2022 (Krogh et al., 2022)	Effects of repetitive transcranial magnetic stimulation on recovery in lower limb muscle strength and gait function following spinal cord injury: a randomized controlled trial	N = 20	Mostly ASIA C-D, one patient with ASIA A	Traumatic and nontraumatic SCI	rTMS vs SHAM	LEMS and gait performance improved significantly at discharge in stimulation group. Not statistically significant results. Trial duration was 4 weeks only.
Lin et al., IEEE Trans Neural Syst Rehabil Eng 2023 (Lin et al., 2023)	Effectiveness of Repetitive Transcranial Magnetic Stimulation Combined With Transspinal Electrical Stimulation on Corticospinal Excitability for Individuals With Incomplete Spinal Cord Injury: A Pilot Study	N = 12	ASIA C-D	no information on aetiology of SCI	iTBS + SHAM vs tsDCS + SHAM vs iTBS + tsDCS vs SHAM + SHAM	Combined therapy showed most promising results during 8 week treatment period.
Nogueira et al., Brain Topogr 2024 (Nogueira et al., 2024)	Repetitive Transcranial Magnetic Stimulation with Body Weight-supported Treadmill Training Enhances Independent Walking of Individuals with Chronic Incomplete Spinal Cord Injury: A Pilot Randomized Clinical Trial	N = 15	ASIA C-D	Mostly traumatic SCI	rTMS + BWSTT vs SHAM + BWSTT	Double-blinded trial which showed LEMS and SCIM-III mobility scores improvements over duration of 4 weeks treatment with rTMS and body weight-support treadmill training (BWSTT). There was no difference in sensory function, functional independence and quality of life between groups.

Belci et al., Spinal Cord 2004 (Belci et al., 2004) not included in this table due to preliminary clinical trial only (N = 4) and no randomisation of subjects.

**Table 2:** List of protocols only for randomised controlled trials.

Author(s), Journal and Year	Title	Patients (N)	SCI grade	Type of SCI	Stimulation type	Functional outcomes
Lacerda de Araújo et al., Trials 2017 (de Araújo et al., 2017)	Effects of high-frequency transcranial magnetic stimulation on functional performance in individuals with incomplete spinal cord injury: **study protocol** for a randomized controlled trial	N = 20	ASIA B-D only	Only traumatic SCI	rTMS vs SHAM	Double-blinded. 4 weeks treatment, with one week for close out. No data.
Sun et al., Neural Regen Res. 2022 (Sun et al., 2022)	Effects of paired associative magnetic stimulation between nerve root and cortex on motor function of lower limbs after spinal cord injury: **study protocol** for a randomized controlled trial.	N = 110	ASIA C-D	Traumatic SCI only	PAS vs SHAM	Blinded (not specified), 4 weeks treatment, 6 months follow up. No data.

Unfortunately, Nogueira et al. showed that there was no difference in patients' quality of life using 36-item short form survey (SF-36) and patient global impression of change scale (PGIS), despite an improvement in patients' LEMS and SCIM III scores after 12 sessions of body weight-support treadmill training (BWSTT) + rTMS (Nogueira et al., 2024). This reiterates the importance of applying suitable functional outcome measures in these studies in order to meaningfully assess the impact on quality of life, albeit that this study only included 15 patients and therefore results have to be interpreted with caution.

### 2.3 Role of nTMS for characterisation of non-traumatic SCI

In a case series with 18 patients by the group in Berlin, Zdunczyk et al. found for the first time that patients with a reduced corticospinal excitability (CE), prolonged cortical silence period (CSP), and restricted motor area presented with more severe symptoms and had less favourable clinical outcomes, compared to those with compensatory recruitment of non-primary motor areas as part of neuroplasticity (Zdunczyk et al., 2018).

Onyiriuka et al. could demonstrate the functional cortical reorganisation in the case of a 67 year-old patient with thoracic intradural extramedullary tumour (meningioma). While pre-operatively no MEPs could be elicited in both lower limbs, one year after surgery the patient had elicitable MEPs with an RMT of 72 % and 65 % in the right and left foot respectively. Cortical activation maps showed recovery of the area and volume of cortical representation of the lower limbs and feet at one year after surgery while the upper limbs showed a reduction in estimated areas and activation volumes (Onyiriuka et al., 2024). The patient had made a reasonable recovery in his lower limb function being independently ambulant for short distances, and his post-operative nTMS correlated with recovery after spinal surgery. This concept is similar to the one applied to using nrTMS for post-surgical stroke patients (Ille et al., 2021), where post-operative patients with present MEPs showed a significant improvement in Fugl-Meyer Assessment and NIHSS scale after nrTMS compared to SHAM-control. Moreover, higher resting motor thresholds (RMTs) pre-operatively seem to indicate a higher risk of post-operative motor deficits (Lavrador et al., 2020).

### 2.4 Limitations and challenges

One of the major challenges when comparing previous studies and review articles on the role of non-invasive brain stimulation in spinal cord injury (Wang et al., 2023; Leszczyńska and Huber, 2023; Awad et al., 2015; Arora et al., 2022; Gunduz et al., 2017; Shulga et al., 2021b; Tazoe and Perez, 2015) is the variability in patient cohort, treatment protocols, and neurological/functional assessments.

There is a substantial logistical challenge in the early application of nTMS in SCI patients that may have to be treated on intensive care for a period of time or that move on to have neuro-rehabilitation. So far, it is unclear what the ideal time-to-nTMS assessment or time-to-nTMS treatment is as well as the length of treatment. Most animal studies have been performed shortly after injury, while in some animal studies they have waited up to 8 weeks and still found meaningful changes (Gunduz et al., 2017; Carmel et al., 2014).

Further drawbacks relate to the inter-user variability and vastly different stimulation protocols that have been applied during clinical trials reducing reproducibility of previous results. Studies using rTMS have shown limited (Benito et al., 2012; Adeel et al., 2022; Nogueira et al., 2024) and sometimes even negative results with regards to improvements in ASIA score or lower limb maximal muscle strength (Krogh et al., 2022; Kuppuswamy et al., 2011; Perez, 2012). However, the data on its use for reducing spasticity and neuropathic pain has been more promising (Wang et al., 2023; Jo and Perez, 2020) but requires further exploration. More recently, researchers have focussed on incorporating different techniques such as PCMS and PAS (Jo and Perez, 2020; Shulga et al., 2021b). Shulga et al. argue that high-PAS can activate weakened connections and may benefit a wide range of patients with incomplete SCI especially those with lower ASIA scores who have been injured more recently (Shulga et al., 2021b). This may be an example where targeting a specific subgroup of patients could lead to better results. Other authors like, Leszynska et al., argue for a combination of electrical and/or magnetic stimulation with kinesiotherapy since the combination of both improves patient neurophysiology and leads to better surface EMG results rather than kinesiotherapy alone (Leszczyńska and Huber, 2023). In fact, in some of the clinical trials mentioned earlier (Adeel et al., 2022; Nogueira et al., 2024) stimulation treatment is combined with physical exercise in the form of bicycling or body weight-support treadmill training. One of the major criticisms of the use of TMS in therapy is the potential waning effect once therapy stops. Most trials that were using TMS for rehabilitation only have a very short follow up period, and hence commenting on the sustenance of treatment effects is difficult. On the other hand, some clinical trials report a relatively high drop-out rate due to time constraints and treatment intensity. It has also been shown that patients suffer from treatment fatigue (Patel et al., 2020), experience mild pain during stimulation which may also impact their quality of life after injury. Regarding the safety of TMS, there have been numerous reports and guidelines stating that it is a safe non-invasive procedure especially with regards to patients with epilepsy (Tarapore et al., 2016; Vucic et al., 2023; Stultz et al., 2020). There are over 30 assessments recommended for use in SCI (Arora et al., 2022) which makes comparisons between functional outcomes challenging as well as they do not necessarily reflect adequately on patient quality of life.

### 2.5 Future directions

The underlying molecular mechanisms of spinal cord injury are poorly understood (Hu et al., 2023) and therefore a reliable, non-invasive tool for prognostication would be useful (Awad et al., 2015; Arora et al., 2022). Some authors argue for early spinal decompression which may improve motor outcome (Punjani et al., 2023; Badhiwala et al., 2021) after spinal cord injury. Badhiwala et al. have performed a pooled analysis of 1548 patient datasets and found that patients undergoing early surgical decompression (within 24 h) made a better recovery than patients undergoing late decompression (Badhiwala et al., 2021; Ellaway et al., 2014). The use of AI and robotics may also assist in better management and prognostication of SCI patients and enables us to identify patient subgroups that will benefit the most for a certain treatment in order to avoid a one size fits all approach. There is a potential for integration of nTMS data, excitability and MEP characteristics, into clinical algorithms as an add-on to the well-established clinical assessment tools to improve patient stratification and prognostication allowing for better treatment and rehabilitation strategies.

Lastly, there may be an interaction of TMS with other experimental, more invasive treatments which could enhance or dampen treatment effects, as an example Tazoe et al. have indicated that Baclofen, which is commonly taken by patients with SCI to reduce spasticity, may interact with the efficacy of rTMS (Tazoe and Perez, 2015). This needs to be considered when planning multifactorial treatment approaches to SCI patients in the future.

In summary, there are some indicative data for the usefulness of nTMS in SCI in the form of clinical trials available however they lack sample size as well as a uniform treatment strategy and outcome measurement in order to allow meaningful interpretation. The previously mentioned protocols (43,44) of randomised controlled trials have not resulted in publication and do not use comparable treatment strategies (rTMS vs SHAM (43) and PAS vs SHAM (44)). In the future, a multicentre randomised controlled trial approach with a uniform and simple treatment strategy (PAS vs SHAM) across all participating centres should be considered. On top of the known functional outcome measurements, quality of life scores should be included and follow up would have to be significantly longer to account for rehabilitation effects.

## 3 Conclusion

TMS offers a useful, non-invasive treatment tool for a holistic approach to SCI and has the potential to improve patient care by being used diagnostically, for prognostication and treatment of patients with traumatic and non-traumatic SCI depending on further validation and elucidation of the published preliminary results.

## Author contributions

J.J. drafted the manuscript. S.P. and A.K. collected demographic, nTMS and clinical data. A.A. and J.P.L helped with conceptualisation of the manuscript. All authors read and approved the final version of this manuscript.

## Declaration of competing interest

The authors declare that they have no known competing financial interests or personal relationships that could have appeared to influence the work reported in this paper.
